# How ecological, production, and living spaces jointly shape urban spatial integration through resource sharing and interaction

**DOI:** 10.3389/fpubh.2025.1651646

**Published:** 2025-09-04

**Authors:** Xiangfei Zhu, Yuqi Liu, Xiaojun Zeng, Yanqing Yan

**Affiliations:** Faculty of Liberal Studies, Guangdong University of Science and Technology, Dongguan, China

**Keywords:** urban spatial integration, structural equation modeling, mediation effect, policy implementation, environmental awareness

## Abstract

This study focuses on the synergistic integration mechanism of urban Production, Living, and Ecological (P–L–E) spaces, employing a structural equation model (SEM) to elucidate their interaction pathways and the moderating effects of policy implementation and public awareness. Based on 750 survey responses from Guangdong Province, the findings indicate that Perceived Quality of Ecological Space (PQES) exerts the strongest influence on the Degree of Urban Spatial Integration (DUSI; *β* = 0.236), followed by Production Space Vitality (PSV) and Satisfaction with Living Space (SLS). The key mediating variables—Degree of Shared Access to Spatial Resources (DSSR) and Frequency of Spatial Interaction (FSI)—serve as critical bridges linking the three spatial dimensions to DUSI, with the indirect effect of PQES via FSI being the most pronounced (*β* = 0.051). Moreover, the Effectiveness of Urban Planning Policy Implementation (EPPI) amplifies the positive impacts of SLS on both resource sharing and interaction frequency, while Public Environmental Awareness (PEA) strengthens the promotive role of ecological space perception in achieving spatial integration. The model explains 43.9% of the variance, unveiling a systematic pathway of “spatial quality enhancement → resource sharing → behavioral interaction → integration achievement.” These findings provide a robust theoretical basis for optimizing the quality of urban production, living, and ecological spaces, as well as for refining policy implementation frameworks. The study underscores the necessity of advancing spatial optimization in tandem with public engagement to build efficient, livable, and sustainable urban spatial systems.

## 1 Introduction

Against the backdrop of rapid global urbanization, the multidimensional integration of urban spatial functions has emerged as a critical issue in the fields of urban planning and governance (1, 2). Cities are not only hubs of economic activity but also serve as vital spaces for human habitation and essential units of the ecological environment (3). However, traditional urban development models have often placed disproportionate emphasis on the expansion of single-function zones—such as industrial areas, residential districts, and ecological reserves—leading to functional imbalances, spatial fragmentation, and inefficient resource utilization (4). Therefore, the synergistic integration of urban Production, Living, and Ecological spaces—hereinafter referred to as the P–L–E ternary space—constitutes a central challenge in advancing sustainable urbanization.

According to national strategic documents such as the National New Urbanization Plan (2014–2020) (5, 6) and the Territorial Spatial Planning Outline (2021–2035) (7), optimizing the spatial layout of production, living, and ecological spaces, and promoting multifunctional, integrated utilization are essential pathways to enhancing urban competitiveness and achieving sustainable development (8, 9). While prior research, grounded in spatial justice theory (10) and resource dependence theory (11), has examined interaction mechanisms between individual spatial dyads (e.g., production–living) (12), a systematic theoretical framework for integrating all three spatial domains remains largely absent.

Two major gaps persist in the literature:

First, prevailing studies tend to focus on the direct influence of spatial quality on integration, neglecting the bridging functions of mediating variables such as resource sharing and behavioral interaction (13, 14).

Second, the moderating roles of policy and public awareness have not been incorporated, constraining our understanding of “government–citizen” collaborative governance mechanisms (15, 16).

To address these gaps, this study develops a comprehensive conceptual framework that integrates spatial quality (PQES, PSV, SLS), resource sharing (DSSR), behavioral interaction (FSI), degree of integration (DUSI), and policy–public moderators (EPPI, PEA). This framework aims to elucidate the underlying systemic logic governing the synergistic integration of P–L–E spaces, thereby advancing both the theoretical and practical frontiers of sustainable urban development.

### 1.1 Theoretical foundation and hypothesis proposition

The role of Production Space Vitality (PSV) in fostering urban functional integration has been extensively and systematically examined in the literature. Zheng et al. reveal that the vibrancy of production spaces acts as a critical catalyst for regional economic resilience by facilitating technological spillovers (17), thereby underpinning the advancement of spatial integration processes. Building on this foundational insight, Zhao et al. empirically substantiate that the strategic optimization of production spaces is a fundamental prerequisite for the realization of coordinated and synergistic urban functions (18). However, prevailing research predominantly concentrates on the dynamic interplay between production and living spaces, often neglecting a comprehensive analysis of PSV’s indirect contributions to the integration of ecological spaces. Anchored in spatial justice theory (19), PSV is conceptualized as a multidimensional driver that not only promotes the equitable allocation of resources essential for living space development but also potentially enhances the qualitative attributes of ecological spaces through the diffusion and application of advanced technological innovations. On this basis, we advance the following hypothesis:

*Hypothesis 1 (H1)*: PSV exerts a positive and substantive influence on the Degree of Urban Spatial Integration (DUSI).

Building upon this, Satisfaction with Living Space (SLS), a core indicator of residents’ well-being, has been extensively validated as closely linked to community cohesion and spatial integration. Du et al. demonstrated a significant positive correlation between living space satisfaction and a sense of community belonging (20), while Khazaie et al. highlighted that the quality of living spaces enhances overall urban functional integration through mediating mechanisms (21–23). Nevertheless, existing research predominantly approaches SLS from a static perspective, insufficiently accounting for its dynamic influence on integration via behavioral interactions such as cross-spatial activity frequency. Grounded in Social Exchange Theory, SLS is posited to foster residents’ engagement, thereby promoting cross-spatial interactions that enhance integration (24–27). Accordingly, we propose:

*Hypothesis 2 (H2)*: SLS exerts a positive effect on the DUSI.

Building on the foregoing, the perceived quality of ecological spaces (PQES) has garnered increasing attention for its impact on residents’ health and spatial integration. Guo et al. identified a significant association between PQES and residents’ psychological well-being (28), while Pang et al. demonstrated that ecological space optimization indirectly facilitates the integration of production and living spaces by enhancing public satisfaction (29). However, extant research predominantly emphasizes the physical attributes of ecological spaces (e.g., green coverage), with limited exploration of the direct mechanisms by which subjective perceptions of ecological space quality influence spatial integration. Grounded in environmental perception and behavior theory, PQES is posited to indirectly promote functional synergy by shaping individuals’ spatial utilization behaviors (30, 31). Therefore, we propose:

*Hypothesis 3 (H3)*: PQES positively influences the DUSI.

Notably, the mediating roles of DSSR and FSI in spatial integration remain insufficiently elucidated. Zhen et al. identified DSSR as a significant mediator in the synergistic relationship between production and living spaces (17), while Guo et al. demonstrated that FSI enhances urban spatial vitality through dynamic interaction mechanisms (32). However, extant studies commonly treat resource sharing and interaction frequency as independent variables, failing to uncover their potential chain-mediating effects linking PSV, SLS, and PQES with DUSI. Grounded in Resource Dependence Theory (33), resource sharing facilitates integration by dismantling spatial barriers, whereas interaction frequency reinforces integration effects via social capital accumulation. Accordingly, we propose:

*Hypothesis 4 (H4)*: DSSR and FSI mediate the relationships between PSV, SLS, PQES, and DUSI.

Moreover, the moderating roles of EPPI and PEA have yet to be adequately incorporated into research on spatial integration. Guo found that policy implementation effectiveness significantly moderates the optimization of spatial resource allocation (34), while Webber et al. indicated that PEA indirectly facilitates ecological space integration through behavioral choices (35). However, existing studies often analyze policy or public factors in isolation, neglecting their synergistic moderating effects on the relationship between spatial quality and integration. Drawing on policy implementation theory and environmental awareness theory, EPPI amplifies the positive effects of spatial quality by enhancing policy enforcement outcomes, whereas PEA strengthens the integration effect of ecological perception by increasing public participation. Accordingly, we propose:

*Hypothesis 5 (H5)*: EPPI and PEA moderate the relationships between PSV, SLS, PQES, and DUSI.

In summary, this study constructs an integrative framework encompassing spatial quality, resource sharing, behavioral interaction, and policy-public moderating factors, proposing a four-stage pathway: spatial quality → resource sharing → behavioral interaction → integration realization. This framework advances the theoretical foundation for sustainable urbanization. Furthermore, the findings offer policymakers a synergistic governance model of “spatial optimization + public participation,” providing data-driven decision support to achieve the goal of “optimizing urban spatial structure” as outlined in the “14th Five-Year Plan for New Urbanization.”

## 2 Materials and methods

### 2.1 Research design

This study employs Structural Equation Modeling (SEM) to systematically analyze the complex path relationships among Production Space Vitality (PSV), Satisfaction with Living Space (SLS), Perceived Quality of Ecological Spaces (PQES), and Degree of Urban Spatial Integration (DUSI). The advantage of SEM lies in its capacity to simultaneously estimate direct and indirect effects among multiple variables, while also validating the reliability and validity of latent constructs—such as “spatial integration”—through factor analysis.

Research Objectives and Regional Selection.

Guangdong Province was selected for this study based on four key rationales:

- Policy Significance: As a pioneering demonstration area under the “14th Five-Year Plan for New Urbanization,” Guangdong explicitly mandates the coordinated development of production, living, and ecological spaces (7), aligning directly with the study’s core objectives.
- Practical Accessibility: The research team’s longstanding presence in Dongguan City enables profound insights into Guangdong’s urban dynamics and facilitates timely acquisition of pivotal policy documents, including the 2023 “Implementation Opinions on Promoting Coordinated Development of Production, Living, and Ecological Spaces.”
- Spatial Diversity: Guangdong manifests pronounced intra-provincial disparities in urbanization levels (e.g., 75% in Guangzhou versus 40% in the northern mountainous regions) and ecological sensitivities (contrasting the Pearl River Delta megacity cluster with northern ecological buffer zones), offering a robust empirical setting to evaluate the model’s applicability and universality.
- Addressing Research Gaps: Prior literature has predominantly examined dyadic spatial interactions, such as production–living or ecological–living relationships. In contrast, Guangdong’s rapid urbanization and experimental policy environment provide an exemplary natural laboratory to rigorously investigate the triadic coordination mechanisms among production, living, and ecological spaces (36).

### 2.2 Instrument

To achieve the research objectives, the researchers designed a structured questionnaire consisting of nine sections. A total of 783 questionnaires were distributed via Wenjuanxing, with all 783 returned, achieving a 100% response rate. During data processing, 23 invalid questionnaires were excluded, leaving 750 valid responses for analysis. The Cronbach’s alpha coefficients for all dimensions exceeded 0.7, indicating good internal consistency for each dimension (See Appendix Tables 1, 2 for details.).

#### 2.2.1 Demographic information

The questionnaire collected basic demographic information from participants, including age, gender, and occupation, comprising a total of 4 items.

#### 2.2.2 PSV

The PSV scale was adapted from the industrial vitality instrument developed by Cavalluzzo et al. (37), comprising 5 items. This scale employs a 5-point Likert rating system, where 1 signifies “Strongly Disagree” and 5 signifies “Strongly Agree.” The Cronbach’s alpha reliability coefficient for the PSV scale is 0.907, demonstrating robust internal consistency.

#### 2.2.3 SLS

The SLS scale is based on the living environment satisfaction questionnaire developed by Prezza and Costantini (38). It encompasses dimensions such as residential environment, community facilities, and neighborhood relations, comprising a total of 6 items. The scale employs a 5-point Likert rating system, where 1 signifies “Extremely Dissatisfied” and 5 signifies “Very Satisfied.” The Cronbach’s alpha reliability coefficient for the SLS scale is 0.895, indicating strong internal consistency.

#### 2.2.4 PQES

PQES was assessed using a scale adapted from Ibrahim (39), incorporating four items that evaluate perceptions of air quality, green landscapes, and ecological stability. Each item was rated on a five-point Likert scale ranging from 1 (“very poor”) to 5 (“very good”). The scale demonstrated strong internal consistency, with a Cronbach’s alpha of 0.878.

#### 2.2.5 DSSR

DSSR was measured using the resource-sharing scale developed by Jiang and Lin (40), which includes four items focusing on the shared utilization of land, public facilities, and information resources. Responses were recorded on a five-point Likert scale. The scale showed good reliability with a Cronbach’s alpha of 0.869.

#### 2.2.6 FSI

FSI was evaluated using a modified version of the spatial interaction questionnaire by Dong et al. (41), consisting of five items covering human mobility, economic transactions, and information exchange. Participants responded using a five-point Likert scale. The instrument yielded a Cronbach’s alpha of 0.876, indicating satisfactory reliability.

#### 2.2.7 EPPI

EPPI was assessed through a four-item scale adapted from Liang et al. (4), aimed at gaging the perceived strength and effectiveness of urban planning policy execution. A five-point Likert scale was applied. The reliability coefficient (Cronbach’s alpha) was 0.829, supporting the scale’s internal consistency.

#### 2.2.8 Pea

PEA was measured using an adapted version of the scale developed by Li et al. (42), which captures individuals’ environmental attitudes, awareness, and pro-environmental behaviors in daily life. The four-item scale employed a five-point Likert format and achieved a Cronbach’s alpha of 0.851, suggesting good reliability.

#### 2.2.9 DUSI

DUSI was operationalized using the urban spatial integration scale developed by Hillier et al. (43), encompassing three key dimensions: functional integration, spatial layout integration, and social integration. The four-item instrument used a five-point Likert scale and exhibited excellent internal consistency (Cronbach’s alpha = 0.886).

### 2.3 Data analysis strategy

Data were analyzed using SPSS 26.0 and SmartPLS 4.1.0.3. Initially, descriptive statistics and correlation analyses were conducted to examine the distribution and interrelationships among variables. Subsequently, Confirmatory Factor Analysis (CFA) was employed to assess the measurement model’s construct validity and internal consistency. Structural Equation Modeling (SEM) was then used to test the hypothesized relationships and investigate the structural pathways, including mediation effects. To enhance the robustness of mediation testing, a bias-corrected bootstrap approach was applied.

## 3 Results

### 3.1 Descriptive analysis

Table 1 illustrates the distribution of key demographic variables, including gender, age, occupation, and years of residence. In terms of gender structure, males accounted for 50.4% and females for 49.6%, presenting a relatively balanced proportion. This balance helps minimize interpretative bias that might stem from gender disparities. Regarding age distribution, the sample predominantly comprises individuals aged between 18 and 59, accounting for 75.1% of the total, with young and middle-aged groups (18–25 years: 30.0%; 26–35 years: 23.1%) forming the core of the surveyed population. These groups are the principal users and participants of urban spaces, making them highly representative for assessing the practical needs and perceived quality of production, living, and ecological spaces. Additionally, 11.3% of the respondents were 60 years or older, whose behavioral preferences and perceptual characteristics regarding ecological space usage contribute essential insights into the evaluation of ecological space quality.

**Table 1:** Demographic characteristics of respondents (*N* = 750).

Construct	Category	Frequency	Percentage (%)	Cumulative (%)
Gender	Male	378	50.4	50.4
	Female	372	49.6	100
Age	Under 18 years	102	13.6	13.6
	18-25 years	225	30	43.6
	26-35 years	173	23.1	66.7
	35-59 years	165	22	88.7
	60 years and above	85	11.3	100
Occupation	Government or public institution employee	74	9.9	9.9
	Educator or researcher	70	9.3	19.2
	Corporate employee	99	13.2	32.4
	Self-employed individual	69	9.2	41.6
	Industrial worker	62	8.3	49.9
	Service industry worker	78	10.4	60.3
	Freelancer	70	9.3	69.6
	Student	157	20.9	90.5
	Others	71	9.5	100
Years of Residence	Less than 1 year	145	19.3	19.3
	1–3 years	155	20.7	40
	3–5 years	135	18	58
	5–10 years	163	21.7	79.7
	More than 10 years	152	20.3	100

As for occupational structure, the survey sample includes government or public institution employees (9.9%), educators and researchers (9.3%), enterprise employees (13.2%), service industry workers (10.4%), self-employed individuals (9.2%), freelancers (9.3%), industrial workers (8.3%), students (20.9%), and other occupational groups (9.5%). This occupational diversity allows for a multi-perspective analysis of differences in satisfaction with living space and perceptions of ecological space quality, thereby enhancing the explanatory power and practical relevance of the variable measurements.

In terms of years of residence, 19.3% of respondents had lived in the city for less than 1 year, 20.7% for 1 to 3 years, 18.0% for 3 to 5 years, 21.7% for 5 to 10 years, and 20.3% for more than 10 years. This indicates a well-balanced representation of both short-term and long-term residents. Such diversity is valuable for analyzing how residential stability influences the Degree of Shared Access to Spatial Resources (DSSR) and Frequency of Spatial Interaction (FSI) and for further exploring their intrinsic relationship with the Degree of Urban Spatial Integration (DUSI).

Overall, the sample demonstrates structural balance and diversity across gender, age, occupation, and residence duration, aligning with the demographic characteristics of urban populations. This enhances the external validity of the study and provides a robust demographic foundation for subsequent analyses of variables such as Production Space Vitality (PSV), Satisfaction with Living Space (SLS), and Perceived Quality of Ecological Space (PQES). More importantly, this demographic diversity enables the research to more comprehensively capture the interactive mechanisms among spatial elements—particularly in examining the mediating pathways through which DSSR and FSI influence DUSI—thereby offering strong explanatory potential and theoretical contributions (Table 2).

**Table 2:** Descriptive statistics of key variables (*N* = 750).

Construct	N	Min	Max	Mean	SD	Skewness	Kurtosis
PSV	750	1	5	3.202	0.987	−0.299	−0.778
SLS	750	1.167	5	3.316	0.874	−0.113	−0.894
PQES	750	1	5	3.541	0.955	−0.361	−0.815
DSSR	750	1	5	3.421	0.936	−0.319	−0.784
FSI	750	1	5	3.413	0.852	−0.253	−0.779
EPPI	750	1	5	3.443	0.901	−0.567	−0.615
PEA	750	1.25	5	3.663	0.882	−0.518	−0.409
DUSI	750	1	5	3.239	0.958	−0.253	−0.695

To comprehensively understand the core elements and interaction mechanisms of urban production, living, and ecological spaces, this study conducted descriptive statistical analyses on the main variables, including minimum, maximum, mean, standard deviation, skewness, and kurtosis. The results indicate that all variables are reasonably distributed, with strong reliability and measurement validity.

From the perspective of central tendency, PEA recorded the highest mean score (M = 3.663, SD = 0.882), reflecting the respondents’ generally strong environmental responsibility and green consciousness. PQES (M = 3.541, SD = 0.955) and DSSR (M = 3.421, SD = 0.936) also showed relatively high levels, suggesting a favorable public perception of the urban ecological environment and the effectiveness of spatial resource-sharing mechanisms.

SLS (M = 3.316, SD = 0.874) and FSI (M = 3.413, SD = 0.852) were at moderately high levels, indicating good accessibility, convenience, and frequency of interpersonal or intergroup interactions in daily urban life. Additionally, the EPPI (M = 3.443, SD = 0.901) was rated above the midpoint, suggesting a certain degree of policy enforcement, though with room for improvement.

In contrast, DUSI (M = 3.239, SD = 0.958) and PSV (M = 3.202, SD = 0.987) were relatively lower, indicating potential for enhancement in the synergetic development of multifunctional urban spaces and in the innovation and dynamism of production systems.

Regarding data distribution, all variables exhibited negative skewness (ranging from −0.113 to −0.567) and negative kurtosis (ranging from −0.409 to −0.894), suggesting slight left-skewness and platykurtic (flatter) distributions. These distributional characteristics imply an absence of extreme skew or peakedness and approximate a normal distribution, which is favorable for subsequent analyses such as structural equation modeling (SEM) and multiple regression.

In summary, the variables in this study are evenly distributed with reliable and representative measurements. This provides a solid empirical foundation for exploring both the direct and indirect effects of PSV, SLS, and PQES on DUSI, as well as for analyzing the mediating and moderating roles of DSSR and FSI within the broader framework of urban spatial integration.

### 3.2 Confirmatory factor analysis

Based on Structural Equation Modeling (SEM), this study conducted a measurement model evaluation of the core variables involved in the “interrelationships and systematic construction of urban production, living, and ecological spaces.”

Table 3 presents the standardized factor loadings for all measurement items. All loadings exceed 0.77, with the highest reaching 0.877, indicating strong convergent validity.

**Table 3:** Factor loadings matrix for measurement indicators of study variables (*N* = 750).

Item	PEA	DUSI	EPPI	PSV	PQES	SLS	FSI	DSSR
DSSR1								0.855
DSSR2								0.823
DSSR3								0.865
DSSR4								0.845
DUSI1		0.847						
DUSI2		0.866						
DUSI3		0.863						
DUSI4		0.877						
EPPI1			0.870					
EPPI2			0.812					
EPPI3			0.772					
EPPI4			0.780					
FSI1							0.837	
FSI2							0.809	
FSI3							0.842	
FSI4							0.802	
FSI5							0.797	
PEA1	0.848							
PEA2	0.831							
PEA3	0.840							
PEA4	0.800							
PQES1					0.842			
PQES2					0.871			
PQES3					0.862			
PQES4					0.847			
PSV1				0.861				
PSV2				0.846				
PSV3				0.861				
PSV4				0.854				
PSV5				0.845				
SLS1						0.817		
SLS2						0.774		
SLS3						0.812		
SLS4						0.825		
SLS5						0.783		
SLS6						0.846		

First, PSV comprises five items, with loadings ranging from 0.845 to 0.861. This indicates a high level of internal consistency, confirming that the construct effectively captures the spatial vibrancy of urban production activities. SLS consists of six indicators, with factor loadings between 0.774 and 0.846, accurately reflecting residents’ perceptions of comfort and satisfaction in their living environment.

Second, all four indicators for PQES exhibit loadings above 0.84, confirming the stable internal structure of this construct in representing residents’ cognitive evaluations of urban ecological systems. Similarly, items under DSSR and FSI all show loadings exceeding 0.80, suggesting strong structural coherence in residents’ behaviors related to resource sharing and interactions across production, living, and ecological domains.

For the mediating and moderating variables, the indicators for EPPI range from 0.772 to 0.870, indicating that respondents demonstrated a high degree of cognitive consistency and discernment regarding the effectiveness of policy implementation. All measurement items for PEA also have loadings above 0.80, highlighting the construct’s significant role in modulating both ecological perceptions and mechanisms of spatial integration.

Finally, the dependent variable DUSI is measured by four indicators with loadings between 0.847 and 0.877. These high values reflect strong construct validity, reinforcing DUSI’s role as a key output variable capturing the integrated state of urban production, living, and ecological spaces.

As shown in Table 4, the Cronbach’s Alpha (*α*) values for all latent constructs range from 0.829 to 0.907, exceeding the recommended threshold of 0.70 (Hair et al., 2019). This indicates that all constructs demonstrate strong internal consistency reliability.

**Table 4:** Convergent validity and construct reliability assessment (*N* = 750).

Construct	Cronbach’s α	Composite reliability (ρₐ)	Composite reliability (ρc)	Average variance extracted (AVE)
PEA	0.851	0.867	0.898	0.688
DUSI	0.886	0.886	0.921	0.745
EPPI	0.829	0.885	0.884	0.655
PSV	0.907	0.909	0.931	0.728
PQES	0.878	0.884	0.916	0.732
SLS	0.895	0.902	0.92	0.656
FSI	0.876	0.881	0.910	0.669
DSSR	0.869	0.871	0.910	0.718

Moreover, the composite reliability (ρₐ and ρc) values for each construct exceed the commonly accepted threshold of 0.70 (44), with several reaching values above 0.90 (e.g., PSV: ρc = 0.931), confirming the high reliability of these measures.

In terms of convergent validity, all constructs have AVE values above the benchmark of 0.50, ranging from 0.655 (EPPI) to 0.745 (DUSI). This suggests that each latent variable explains more than half of the variance in its observed indicators, meeting the criteria for adequate convergent validity.

Specifically:

- The AVE for PEA is 0.688, indicating that the construct successfully captures the variance in environmental cognition and behavioral awareness across respondents.
- DUSI, as the core dependent variable, shows the highest AVE (0.745), supporting its strong convergent structure in measuring urban spatial integration.
- PSV and PQES also exhibit excellent convergence (AVE > 0.72), reflecting the internal coherence of spatial vitality and ecological perception constructs.
- SLS and DSSR maintain both reliability (CR > 0.90) and acceptable AVE (> 0.65), ensuring the robustness of social-perceptual measurements within the urban space triad.

Table 5 reports the square roots of the AVE along the diagonal and the inter-construct correlation coefficients in the off-diagonal cells, based on the Fornell-Larcker criterion for assessing discriminant validity.

**Table 5:** Matrix of AVE square roots and correlation coefficients for latent variables (Fornell-Larcker discriminant validity; *N* = 750).

Construct	PEA	DUSI	EPPI	PSV	PQES	SLS	FSI	DSSR
PEA	0.83							
DUSI	0.067	0.863						
EPPI	0.256	−0.034	0.809					
PSV	0.061	0.45	0.093	0.854				
PQES	0.055	0.432	0.03	0.34	0.856			
SLS	0.024	0.402	0.021	0.296	0.299	0.81		
FSI	0.112	0.472	−0.047	0.346	0.362	0.331	0.818	
DSSR	0.046	0.444	−0.061	0.344	0.322	0.304	0.358	0.847

According to the Fornell-Larcker criterion (45), discriminant validity is established when the square root of a construct’s AVE exceeds its correlations with all other latent constructs. The results in Table 5 demonstrate that each diagonal value (√AVE) is higher than any of the corresponding off-diagonal correlations for each construct.

For example, the square root of AVE for Degree of Urban Spatial Integration (DUSI) is 0.863, which is greater than its highest correlation with any other construct (e.g., 0.472 with Frequency of Spatial Interaction, FSI), supporting the distinctiveness of DUSI as a latent factor. Similarly, Production Space Vitality (PSV) demonstrates adequate discriminant validity, with √AVE = 0.854, exceeding its highest inter-construct correlation (0.450 with DUSI).

Even constructs with moderate inter-correlations, such as FSI and DSSR (*r* = 0.358), still meet the discriminant validity criterion, as their respective √AVE values (0.818 and 0.847) remain higher than the shared variance. These results indicate that the constructs in this study are empirically distinct from each other and measure unique theoretical dimensions within the urban spatial system.

In summary, the measurement model exhibits satisfactory discriminant validity based on the Fornell-Larcker criterion, ensuring that the latent constructs do not exhibit excessive overlap and are thus appropriate for inclusion in subsequent structural path modeling analyses.

The Heterotrait-Monotrait ratio (HTMT) is a widely recognized method for evaluating discriminant validity in variance-based structural equation modeling, particularly in PLS-SEM frameworks. According to Henseler et al. (46), HTMT values should ideally be below 0.85 (conservative threshold), while values under 0.90 are also considered acceptable in more liberal evaluations.

From Table 6, it can be observed that

- All HTMT values fall below 0.60, with the highest observed value being 0.533 (between DUSI and FSI), which is well below the 0.85 threshold.
- Low HTMT values, such as 0.039 (PEA–SLS), 0.044 (EPPI–SLS), and 0.049 (EPPI–PQES), indicate a clear empirical distinction between the latent constructs involved.

The strongest associations are observed among variables within similar spatial dimensions:

- DUSI–FSI (0.533) and DUSI–DSSR (0.506) suggest that spatial integration is strongly and perceptibly related to spatial interaction and resource-sharing mechanisms.
- PQES–FSI (0.407) and PQES–DSSR (0.365) also reflect the functional relationship between environmental perceptions and spatial social mechanisms.

**Table 6:** Discriminant validity: Heterotrait-Monotrait ratio (HTMT; *N* = 750).

Construct	PEA	DUSI	EPPI	PSV	PQES	SLS	FSI	DSSR
PEA	–							
DUSI	0.074	–						
EPPI	0.312	0.044	–					
PSV	0.071	0.5	0.111	–				
PQES	0.07	0.487	0.049	0.377	–			
SLS	0.039	0.449	0.044	0.323	0.331	–		
FSI	0.127	0.533	0.052	0.385	0.407	0.365	–	
DSSR	0.056	0.506	0.066	0.386	0.365	0.34	0.408	–

These findings strongly support the discriminant validity of all constructs in the model.

Variance Inflation Factor (VIF) is a key diagnostic indicator to detect multicollinearity in regression and structural equation modeling. A VIF value greater than 5 suggests moderate collinearity, and values exceeding 10 are commonly regarded as indicating serious multicollinearity (47, 48). However, in PLS-SEM, a conservative threshold of 3.3 is often adopted to ensure model robustness.

Based on Table 7, all VIF values are comfortably below the critical threshold of 3.3:

- The highest VIF is 1.466 (for PQES), still indicating low multicollinearity.
- Most values fall between 1.01 and 1.40, signifying stable and independent explanatory power of the latent variables.
- For instance, PEA’s influence on FSI and DSSR has VIF values of 1.083, indicating negligible collinearity concerns.

**Table 7:** Collinearity statistics (Variance inflation factor: VIF; *N* = 750).

Construct	PEA	DUSI	EPPI	PSV	PQES	SLS	FSI	DSSR
PEA		1.015					1.083	1.083
DUSI								
EPPI							1.1	1.1
PSV		1.309					1.349	1.349
PQES		1.466					1.323	1.323
SLS		1.233					1.202	1.202
FSI		1.403						
DSSR		1.377						

### 3.3 Bivariate correlation analysis

The Pearson correlation analysis (Table 8) reveals several significant positive relationships among the key constructs in this study:

- PSV is positively and significantly correlated with all other core constructs, especially DUSI (*r* = 0.449, *p* < 0.01), FSI (*r* = 0.345, *p* < 0.01), and DSSR (*r =* 0.343, *p* < 0.01), indicating that greater vibrancy in production space is associated with stronger urban spatial integration and more frequent inter-spatial interactions.
- SLS also demonstrates significant positive correlations with DUSI (*r* = 0.401), FSI (*r* = 0.327), and DSSR (*r* = 0.301), supporting the idea that residential satisfaction is linked to better spatial cohesion and engagement across functional zones.
- PQES is significantly correlated with all major constructs except EPPI, with strong associations to FSI (*r* = 0.359) and DUSI (*r* = 0.430). This underscores the role of environmental perceptions in promoting spatial integration.
- FSI and DSSR show a strong interrelationship (*r* = 0.358, *p* < 0.01) and are both significantly related to DUSI, further validating their roles as mediating variables in the integrated spatial framework.
- EPPI shows only a weak correlation with PSV (*r* = 0.096) and has nonsignificant or slightly negative correlations with most other constructs, suggesting a limited direct influence within the scope of this study’s bivariate analysis.
- PEA shows a modest but significant correlation with FSI (*r* = 0.108, *p* < 0.01) and a moderate correlation with EPPI (*r* = 0.262), which may indicate that public environmental consciousness is more aligned with broader policy awareness than direct spatial engagement.
- The strongest correlations with DUSI are observed for FSI (*r* = 0.471), PQES (*r* = 0.430), and PSV (*r* = 0.449), reinforcing the structural hypothesis that production vibrancy, ecological quality, and spatial interaction jointly facilitate urban spatial integration.

**Table 8:** Bivariate correlation analysis (Pearson’s r; *N* = 750).

Construct	Mean	PSV	SLS	PQES	DSSR	FSI	EPPI	PEA	DUSI
PSV	3.202	1							
SLS	3.316	0.293**	1						
PQES	3.541	0.338**	0.295**	1					
DSSR	3.421	0.343**	0.301**	0.319**	1				
FSI	3.413	0.345**	0.327**	0.359**	0.358**	1			
EPPI	3.443	0.096**	0.025	0.032	−0.056	−0.046	1		
PEA	3.663	0.062	0.026	0.058	0.047	0.108**	0.262**	1	
DUSI	3.239	0.449**	0.401**	0.430**	0.444**	0.471**	−0.036	0.064	1

Correlation is significant at the 0.01 level (2-tailed).

### 3.4 Structural equation modeling analysis

**Figure 1:**
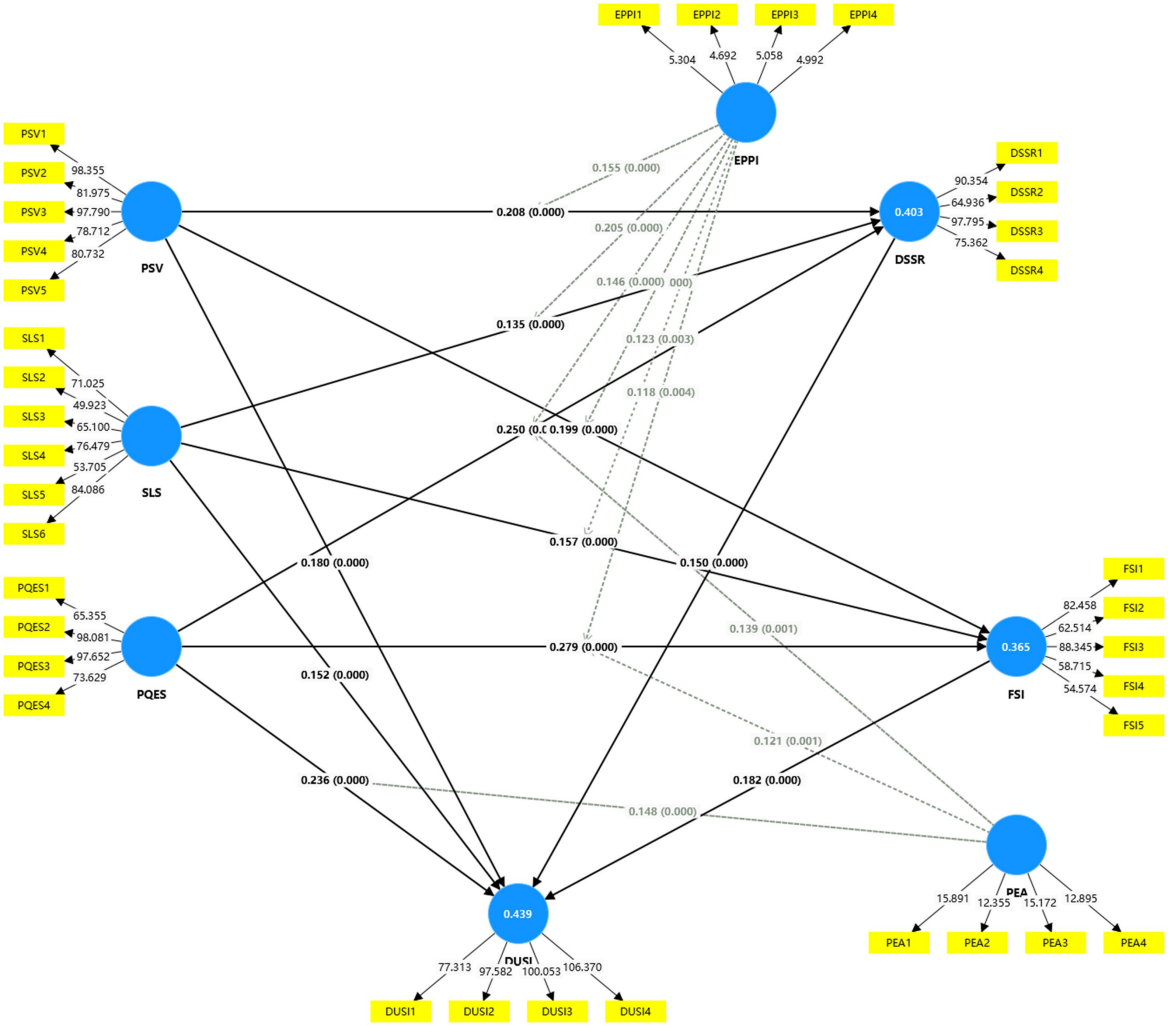
Structural equation model using SmartPLS (*N* = 750).

To further investigate the interrelationships among constructs and test the proposed hypotheses, a Structural Equation Modeling approach was employed using SmartPLS 4.1.0.3. The measurement and structural model are depicted in Figure 1. The model incorporates both direct and indirect pathways from latent exogenous variables—PSV, SLS, PQES, EPPI, and PEA—to the endogenous outcome, DUSI, mediated by DSSR and FSI.

As shown in Table 9, all hypothesized structural paths within the model demonstrate a high level of statistical significance (*p* < 0.001), indicating robust and reliable relationships among the latent variables. The following findings merit particular attention:

**Table 9:** Path coefficient analysis based on PLS-SEM results (*N* = 750).

Path	*O*	*M*	*Std.*	*T*	*p*
PSV→DUSI	0.18	0.18	0.035	5.138	0.000
PSV→FSI	0.199	0.196	0.036	5.486	0.000
PSV→DSSR	0.208	0.205	0.035	5.876	0.000
PQES→DUSI	0.236	0.234	0.037	6.461	0.000
PQES→FSI	0.279	0.277	0.036	7.71	0.000
PQES→DSSR	0.25	0.25	0.035	7.174	0.000
SLS→DUSI	0.152	0.154	0.033	4.606	0.000
SLS→FSI	0.157	0.158	0.034	4.573	0.000
SLS→DSSR	0.135	0.136	0.035	3.907	0.000
FSI→DUSI	0.182	0.182	0.037	4.945	0.000
DSSR→USI	0.15	0.15	0.037	3.99	0.000

Among the three core dimensions, PQES exerts the most substantial impact across the model. Specifically, its effects on both FSI (*β* = 0.279, T = 7.710) and DSSR (*β* = 0.250, T = 7.174) are the strongest observed, suggesting that improvements in the perceived ecological quality can significantly enhance the frequency of interpersonal or intergroup interactions and promote broader spatial co-utilization. Additionally, PQES contributes directly to DUSI (*β* = 0.236, T = 6.461), highlighting its pivotal role not only as an environmental metric but also as a catalyst for urban spatial cohesion.

PSV also exhibits statistically significant influence on DUSI (*β* = 0.180, T = 5.138), while indirectly enhancing it through its positive effects on both FSI (*β* = 0.199) and DSSR (*β* = 0.208). These results underscore that vibrant, multifunctional production spaces foster dynamic interactions and resource-sharing behaviors among urban actors, thereby laying a functional foundation for integrated spatial development.

While the effects of SLS are comparatively modest, they remain significant. SLS positively affects DUSI (*β* = 0.152, T = 4.606), FSI (*β* = 0.157, T = 4.573), and DSSR (*β* = 0.135, T = 3.907). These findings indicate that improved satisfaction with residential environments can meaningfully contribute to overall spatial integration, primarily by reinforcing residents’ sense of spatial belonging and their willingness to engage in shared spatial practices.

Notably, two mediating mechanisms also demonstrate statistical significance:

- FSI → DUSI (*β* = 0.182, *t* = 4.945)
- DSSR → DUSI (*β* = 0.150, *t* = 3.990)

These pathways confirm that DUSI is not solely determined by subjective evaluations such as PQES and SLS but is also critically mediated by the concrete dynamics of spatial behavior—namely, how frequently space users interact and to what extent spatial resources are shared. From a spatial governance perspective, this underscores the importance of fostering “interaction-based and resource-sharing-driven integration” as a strategic pathway toward achieving cohesive urban spatial systems.

The results of the moderation analysis (see Table 10) indicate that both EPPI and PEA play significant moderating roles in the structural relationships among core spatial variables, with all interaction effects reaching statistical significance (*p* < 0.01).

**Table 10:** Moderation effects analysis (*N* = 750).

Path	*O*	*M*	*Std.*	*T*	*p*
EPPI × SLS → FSI	0.123	0.116	0.042	2.955	0.003
EPPI × SLS → DSSR	0.205	0.191	0.047	4.383	0.000
PEA × PQES → DUSI	0.148	0.146	0.033	4.461	0.000
PEA × PQES → FSI	0.121	0.127	0.038	3.178	0.001
PEA × PQES → DSSR	0.139	0.147	0.041	3.386	0.001
EPPI × PSV → FSI	0.151	0.144	0.043	3.507	0.000
EPPI × PSV → DSSR	0.155	0.145	0.04	3.878	0.000
EPPI × PQES → FSI	0.118	0.111	0.042	2.853	0.004
EPPI × PQES → DSSR	0.146	0.14	0.041	3.584	0.000

First, EPPI significantly moderates the relationship between SLS and two key mediators:

- EPPI × SLS → FSI: *β* = 0.123, *t* = 2.955, *p* = 0.003
- EPPI × SLS → DSSR: *β* = 0.205, *t* = 4.383, *p* < 0.001

These results suggest that the influence of residents’ satisfaction with their living environments on their participation in spatial interaction and resource-sharing practices is significantly strengthened under more effective urban policy implementation. In other words, high EPPI levels amplify the behavioral activation effects of SLS.

Furthermore, PEA acts as a significant moderator in the pathways from PQES to all three outcome variables:

- PEA × PQES → DUSI: *β* = 0.148, *t* = 4.461, *p* < 0.001
- PEA × PQES → FSI: *β* = 0.121, *t* = 3.178, *p* = 0.001
- PEA × PQES → DSSR: β = 0.139, *t* = 3.386, *p* = 0.001

This finding indicates that citizens’ environmental awareness not only enhances their ecological perceptions but also reinforces how these perceptions translate into urban integration, interaction, and co-utilization behaviors. Thus, environmental education and awareness-building campaigns can serve as critical leverage points in ecological governance.

In addition, EPPI also strengthens the influence of PSV and PQES on mediators:

- EPPI × PSV → FSI: *β* = 0.151, *t* = 3.507, *p* < 0.001
- EPPI × PSV → DSSR: *β* = 0.155, *t* = 3.878, *p* < 0.001
- EPPI × PQES → FSI: *β* = 0.118, *t* = 2.853, *p* = 0.004
- EPPI × PQES → DSSR: *β* = 0.146, *t* = 3.584, *p* < 0.001

Collectively, these moderation effects illustrate that urban planning governance and citizen-level environmental cognition are not merely contextual variables but function as critical enablers that amplify the transmission mechanisms from space perception/satisfaction to behavioral outcomes. The findings underscore the need for integrated planning frameworks that combine top-down policy effectiveness with bottom-up public awareness to fully realize spatial synergy and urban integration.

The R-square (R^2^) values (Table 11) of the structural model offer insight into the model’s explanatory power for the three endogenous constructs: DUSI, FSI, and DSSR.

- The model explains 43.9% of the variance in DUSI (*R^2^ = 0.439; Adjusted R^2^ = 0.434*), suggesting a moderate to substantial level of explanatory power for how spatial integration emerges as a composite outcome of production, ecological, and living space attributes, moderated by planning and awareness factors.
- For FSI, the model accounts for 36.5% of the variance (*R^2^ = 0.365; Adjusted R^2^ = 0.357*), indicating a moderate degree of predictability. This implies that while predictors such as Production Space Vitality (PSV), Satisfaction with Living Space (SLS), and Perceived Quality of Ecological Space (PQES) significantly contribute to explaining interaction frequency, other unmeasured behavioral or infrastructural variables may still play a role.
- The DSSR model explains 40.3% of the variance (*R^2^ = 0.403; Adjusted R^2^ = 0.396*), reflecting a robust explanatory strength. This suggests that the integration of urban production, living, and ecological dimensions—along with moderating effects from policy implementation and public environmental awareness—contributes meaningfully to the extent of spatial resource co-utilization.

**Table 11:** Explained variance (R^2^) analysis of endogenous construct (*N* = 750).

Construct	R-squared	Adjusted R-squared
DUSI	0.439	0.434
FSI	0.365	0.357
DSSR	0.403	0.396

These R^2^ values are consistent with benchmarks in urban spatial modeling literature (e.g., Hair et al., 2020), where values between 0.25 and 0.50 typically represent moderate explanatory strength, especially in complex models involving multidimensional constructs and latent interactions. Hence, the model demonstrates adequate predictive relevance, particularly for urban spatial integration outcomes.

To further evaluate the relative impact of each exogenous construct on the endogenous variables, Cohen’s f^2^ effect sizes were computed. According to Cohen’s guidelines (49), an f^2^ of 0.02 is considered small, 0.15 is medium, and 0.35 is large. The findings from Table 12 reveal that most path relationships exhibit small but meaningful effect sizes, aligning with the multifactorial nature of urban spatial systems.

**Table 12:** Effect size (*f*^2^) analysis of predictor variables (*N* = 750).

Path	*F* ^2^
PSV → DUSI	0.044
PSV → FSI	0.046
PSV → DSSR	0.053
PQES → DUSI	0.068
PQES → FSI	0.093
PQES → DSSR	0.079
SLS → DUSI	0.033
SLS → FSI	0.032
SLS → DSSR	0.026
FSI → DUSI	0.042
DSSR → DUSI	0.029
EPPI × SLS → FSI	0.022
EPPI × SLS → DSSR	0.065
PEA × PQES → DUSI	0.037
PEA × PQES → FSI	0.022
PEA × PQES → DSSR	0.032
EPPI × PSV→FSI	0.034
EPPI × PSV→DSSR	0.037
EPPI × PQES→FSI	0.021
EPPI × PQES→DSSR	0.035

Among all the core predictors, PQES exhibits the strongest set of direct effects. Specifically, the effect of PQES on FSI reaches an f^2^ value of 0.093, indicating a relatively notable explanatory power. Furthermore, its effect on DSSR is marked by an f^2^ of 0.079, while the effect on DUSI yields an f^2^ value of 0.068. These values collectively suggest that PQES plays a leading role in influencing the three key endogenous variables in the model.

These small-to-moderate effects highlight the critical role of ecological spatial perception in driving interaction, sharing, and integration.

In addition to PQES, PSV also exhibits a consistent, albeit slightly lower, level of direct influence on the key outcome variables. Specifically, PSV yields an f^2^ value of 0.053 for its effect on DSSR, 0.046 for FSI, and 0.044 for DUSI. These values indicate that PSV remains a meaningful contributor across all three domains.

By contrast, SLS demonstrates the weakest set of direct effects among the core predictors. Its impact on DUSI is reflected in an f^2^ of 0.033, while its effects on FSI and DSSR are 0.032 and 0.026, respectively. These results suggest that although SLS exerts statistically significant effects, its relative influence is noticeably less substantial compared to that of PQES and PSV.

Regarding the mediating variables, both FSI and DSSR exert meaningful influence on DUSI. The f^2^ values for their effects are 0.042 (FSI → DUSI) and 0.029 (DSSR → DUSI), respectively. These findings indicate that while both variables act as mediators, FSI plays a slightly more prominent mediating role in enhancing urban spatial integration compared to DSSR.

Moderation Effects:

- Among moderating variables, the interaction between EPPI and SLS on DSSR exhibits the highest f^2^ (0.065), reflecting a notable synergistic influence on shared access when both policy strength and living satisfaction are high.
- Other moderation effects, such as EPPI × PQES on DSSR (f^2^ = 0.035) and EPPI × PSV on FSI (f^2^ = 0.034), also indicate meaningful, albeit modest, conditional effects.
- Interactions involving PEA, particularly PEA × PQES, show f^2^ ranging from 0.022 to 0.022–0.037, reinforcing the argument that public engagement with ecological quality moderately enhances integration and interaction outcomes.

Collectively, the f^2^ results affirm that while no single predictor exhibits a large standalone effect, PQES and PSV consistently yield stronger influence than SLS. Moreover, the moderating roles of EPPI and PEA suggest that effective policy implementation and heightened environmental awareness can amplify the effects of spatial quality perceptions on urban integration. These findings underscore the need for holistic urban governance frameworks that integrate physical space optimization with behavioral and policy dimensions.

Table 13 summarizes the results of the predictive relevance (Q^2^) test, obtained via the blindfolding procedure. The Q^2^ statistic measures the model’s capacity to predict the endogenous constructs, with a value greater than zero indicating the presence of predictive relevance (50).

**Table 13:** Predictive relevance (Q^2^) of endogenous constructs (*N* = 750).

Construct	SSO	Sum of squared errors (SSE)	Q^2^(=1-SSE/SSO)
PEA	3,000	3,000	0.000
DUSI	3,000	2029.432	0.324
EPPI	3,000	3,000	0.000
PSV	3,750	3,750	0.000
PQES	3,000	3,000	0.000
SLS	4,500	4,500	0.000
FSI	3,750	2860.919	0.237
DSSR	3,000	2147.906	0.284

The construct DUSI demonstrates the strongest predictive relevance, with a Q^2^ value of 0.324, suggesting that the model substantially explains the variance in this dimension of spatial cohesion. Similarly, the constructs FSI and DSSR yield Q^2^ values of 0.237 and 0.284, respectively, indicating moderate but meaningful predictive relevance. These findings confirm that the model is effective in predicting key urban spatial outcomes.

In contrast, the constructs PEA, EPPI, PSV, PQES, and SLS all report Q^2^ values of 0.000. This indicates that the structural model does not aim to predict these exogenous variables, and thus they do not exhibit predictive relevance under the Q^2^ criterion.

Table 14 presents the specific indirect effects of three core spatial constructs—PSV, PQES, and SLS—on the DUSI, mediated through two intermediate variables: FSI and DSSR. The results are derived from a bootstrapping procedure based on 5,000 resamples, providing robust estimates of the indirect paths.

**Table 14:** Specific indirect effects (*N* = 750).

Path	*O*	*M*	*Std.*	*T*	*p*
PSV → DSSR → DUSI	0.031	0.031	0.009	3.339	0.001
PSV → FSI → DUSI	0.036	0.036	0.01	3.665	0.000
PQES → DSSR → DUSI	0.037	0.037	0.01	3.569	0.000
PQES → FSI → DUSI	0.051	0.051	0.012	4.078	0.000
SLS → DSSR → DUSI	0.02	0.021	0.008	2.621	0.009
SLS → FSI → DUSI	0.029	0.029	0.009	3.279	0.001

The values are based on bootstrapping with 5,000 resamples.

The findings reveal several noteworthy patterns:

1. PSV exhibits statistically significant indirect effects on DUSI through both mediators. Specifically, PSV → DSSR → DUSI (*β* = 0.031, *p* = 0.001) and PSV → FSI → DUSI (*β* = 0.036, *p* < 0.001) indicate that both shared spatial resources and interaction frequency serve as meaningful transmission mechanisms through which production-related spatial dynamics contribute to urban spatial integration.
2. PQES demonstrates the strongest mediating effects among the three predictors. The pathways PQES → DSSR → DUSI (*β* = 0.037, *p* < 0.001) and PQES → FSI → DUSI (*β* = 0.051, *p* < 0.001) highlight that ecological perceptions significantly enhance urban spatial integration via both resource sharing and social interaction frequency. Notably, the PQES → FSI → DUSI path displays the highest indirect effect observed in the table, underscoring the salience of ecological quality in fostering interaction-led spatial cohesion.
3. SLS also contributes significantly to DUSI through both mediators, though the magnitudes are comparatively smaller. The indirect effects SLS → DSSR → DUSI (*β* = 0.020, *p* = 0.009) and SLS → FSI → DUSI (*β* = 0.029, *p* = 0.001) indicate that residential satisfaction does play a role in urban spatial integration, albeit with a more modest impact relative to ecological and production factors.

Overall, the results support a nuanced mediation model in which FSI and DSSR act as crucial conduits between foundational spatial dimensions (i.e., production, ecological, and living space characteristics) and the ultimate integration of urban space. Among the mediators, FSI appears to be slightly more influential than DSSR, particularly in the context of ecological perceptions. These findings underscore the importance of enhancing both interaction opportunities and shared resource accessibility to promote more integrated and cohesive urban environments.

**Figure 2:**
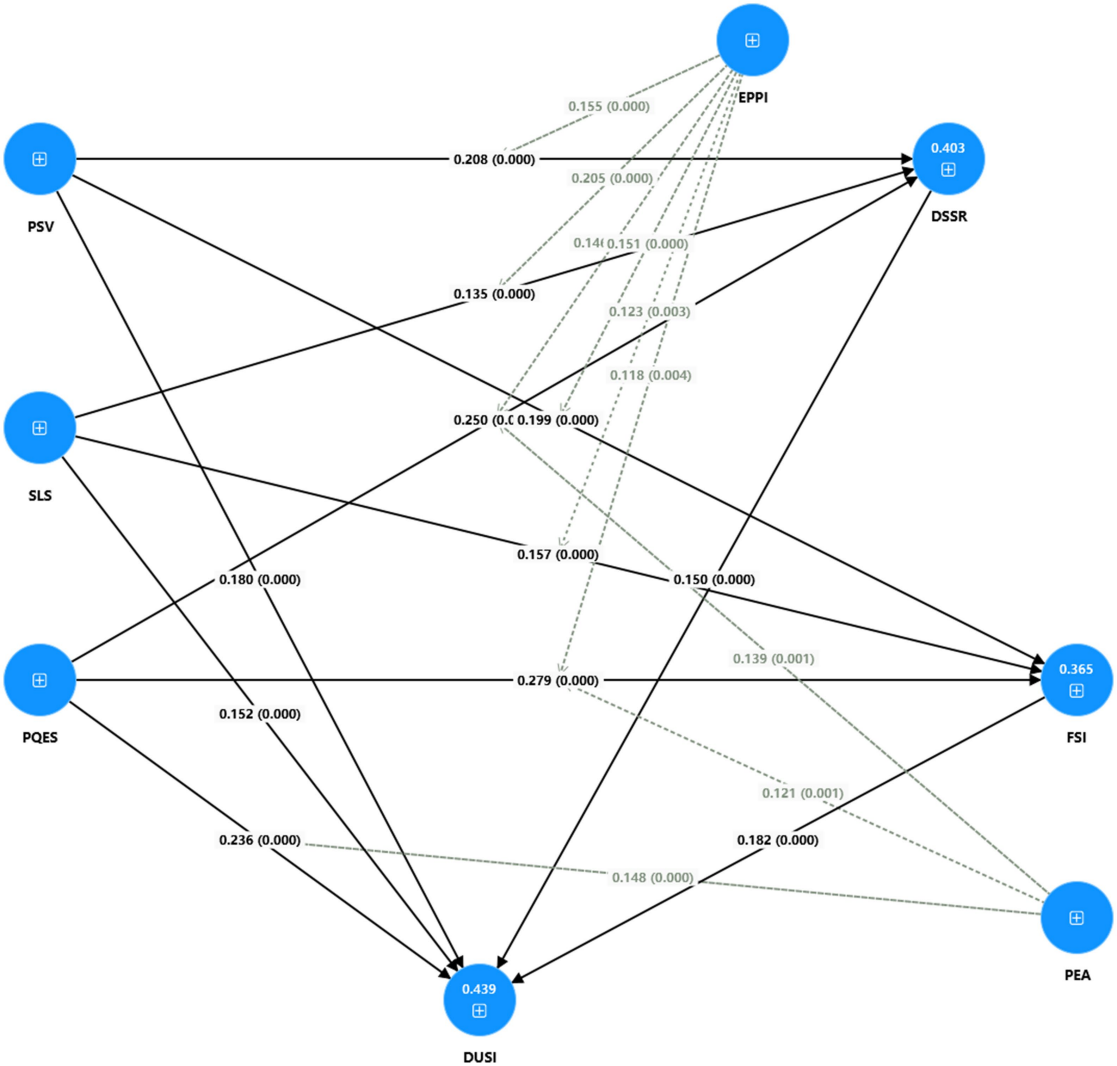
Interrelationship and system modeling of urban production, living and ecological spaces.

Based on the data analysis in the above table, the final model diagram can be obtained, see Figure 2.

## 4 Discussion

### 4.1 Contributions to the literature and theoretical advances

This study makes a substantial contribution to urban spatial integration theory by establishing a comprehensive, behaviorally mediated model linking production space vitality (PSV), satisfaction with living space (SLS), and perceived quality of ecological space (PQES) to the degree of urban spatial integration (DUSI), via the dual mediating channels of shared access to spatial resources (DSSR) and frequency of spatial interaction (FSI). While extant studies have often explored binary spatial dynamics, such as production-living (36) or ecological-living interactions (51), few have addressed the systemic interplay of all three—production, living, and ecological dimensions (P-L-E spaces)—within a unified analytical framework.

Notably, our findings reposition PQES as the most critical driver of urban integration, showing both the strongest direct influence on DUSI and the most robust indirect effect through FSI. This contradicts the traditional emphasis on infrastructural or economic variables (52, 53) and instead elevates subjective ecological perception as a foundational element in integration processes. While Faraz et al. (54) emphasized environmental perception in shaping behavior, our study demonstrates how PQES operates through FSI and DSSR, translating environmental cognition into system-level spatial integration outcomes.

The dual mediation effect of DSSR and FSI is another novel contribution. Existing literature has often treated shared resource access and social interaction as independent facilitators (55, 56). In contrast, our study empirically tests and supports a chain-mediated pathway, highlighting the sequential role of sharing in fostering interaction, which subsequently leads to spatial integration. This sequencing supports the logic of Jiang et al.(2023) resource dependence theory in an urban governance context (33).

Moderating effects of EPPI and PEA further extend prior research. By incorporating both top-down (policy) and bottom-up (citizen awareness) mechanisms into the analytical model, the study offers a more nuanced view of how governance and civic participation shape urban space dynamics. Digdoyo et al. stressed the significance of civic awareness in promoting ecological behavior (57); our findings go further to demonstrate its synergistic effect with perceived ecological quality on integration outcomes.

### 4.2 Interpretation of core findings in scholarly context

The elevation of PQES over PSV and SLS in determining urban spatial integration marks a paradigmatic shift. Traditionally, urban planning has focused on production capacity and residential infrastructure. Our results suggest that perceived ecological quality—though often overlooked—plays a primary role in driving citizen engagement and cross-boundary spatial behaviors. This calls into question the adequacy of traditional infrastructure-first urban policies and aligns more closely with emerging discourses in green urbanism and subjective environmental well-being (58).

The relatively modest direct effect of SLS on DUSI adds nuance to the literature. While past studies have linked residential satisfaction with cohesion and stability (59), our findings suggest that satisfaction alone is insufficient to drive broader spatial integration. It requires activation through mechanisms like shared use and intergroup interaction, a view that aligns with social exchange theory (60) but has not been empirically tested at this scale.

In contrast, PSV’s influence appears conditional, heavily mediated by DSSR and FSI. This implies that economic vitality or industrial clustering, in isolation, does not enhance integration unless paired with inclusive policies that foster sharing and cross-sectoral engagement. Thus, spatial design for economic zones should also incorporate participatory and community-access components.

Lastly, our results underscore the contingent nature of integration outcomes, shaped significantly by EPPI and PEA. Their moderating effects indicate that similar physical environments can yield divergent integration outcomes depending on policy effectiveness and citizen engagement levels. This calls for a governance-sensitive model of urban spatial development.

### 4.3 Empirical contributions and methodological innovation

Methodologically, this study offers a robust structural equation model (SEM) incorporating both mediation and moderation effects. The model explains 43.9% of the variance in DUSI—a considerable improvement over traditional two-domain models. This affirms the value of integrating perceptual, behavioral, and structural variables into one analytical schema. Furthermore, the operationalization of PQES as a subjective construct, rather than merely relying on objective ecological indicators, aligns with best practices in environmental psychology but is rarely seen in urban planning models.

The dual-path mediation and cross-variable moderation reflect a more complex urban reality and provide a replicable framework for future studies. The use of DSSR and FSI as sequential mediators, and the inclusion of both policy execution (EPPI) and citizen behavior (PEA) as moderators, demonstrate a sophisticated design capable of capturing the multi-layered nature of urban integration.

### 4.4 Practical implications

To facilitate urban spatial integration, policymakers and urban planners should prioritize perceptual and behavioral dimensions alongside infrastructural development:

- Ecological Design and Perception Management: PQES’s significant influence suggests a need to enhance not just the physical attributes of ecological spaces but also how citizens perceive and engage with them. Programs promoting community gardening, participatory design, or ecological storytelling can strengthen this link.
- Resource Sharing Mechanisms: The role of DSSR implies that spatial integration thrives where institutional arrangements support access equity. This includes open-access policies, shared-use agreements, and cross-sector spatial coordination.
- Interaction Facilitation: FSI as a key pathway highlights the need for spatial designs and governance strategies that promote movement, exchange, and collaboration across spatial domains. This could involve mixed-use zoning, public events in hybrid spaces, or mobility infrastructure connecting residential, production, and ecological areas.
- Governance Execution and Civic Engagement: The moderating roles of EPPI and PEA point to a dual imperative: effective implementation of integration-friendly policies and citizen empowerment through education and participatory governance structures.

### 4.5 Theoretical implications

This study advances urban spatial integration theory in three keyways:

- From Binary to Triadic Integration Models: Most previous research considers dyadic relationships (e.g., living-ecological). We introduce a triadic, system-level model that better reflects urban complexity.
- Behavioral Mediation over Structural Determinism: The study shifts the analytical focus from static structural indicators to dynamic mediators—sharing and interaction—underscoring the behavioral mechanisms through which space qualities translate into system outcomes.
- Policy and Perception as Conditional Enablers: By validating EPPI and PEA as moderators, the study embeds urban integration within broader governance and cultural contexts, challenging universalist assumptions in spatial planning theory.

### 4.6 Directions for future research

Based on the study’s findings and limitations, we recommend the following targeted future research directions:

- Experimental Interventions in PQES Perception: Since PQES emerged as the strongest predictor of DUSI, future studies should test causal interventions that enhance ecological perception—such as augmented reality ecological layers or citizen-led ecological assessments—to evaluate their effect on integration pathways.
- Cross-regional Comparative Validation: To test the model’s external validity, researchers should apply it in cities with varying levels of ecological endowment, governance quality, and civic engagement. This can reveal boundary conditions for the observed effects.
- Qualitative Studies on EPPI and PEA: The moderating effects of policy and awareness deserve deeper exploration. Future research can deploy interviews, focus groups, or ethnographic methods to understand how citizens perceive policy execution and environmental narratives and how these perceptions shape sharing and interaction behaviors.

## 5 Conclusion

This study proposes and validates a novel theoretical framework explaining how the vitality of production space, satisfaction with living space, and perceived quality of ecological space collectively influence urban spatial integration through two key mediators—spatial resource sharing and spatial interaction frequency. The research confirms that ecological perception, often undervalued in urban planning, plays a central role in shaping integrative spatial behaviors and outcomes.

Importantly, the study reveals that the same spatial qualities can yield divergent integration results depending on the effectiveness of policy execution and the level of public environmental awareness. This underscores the need for cities to adopt a dual strategy: improving the perceptual and functional qualities of urban spaces while simultaneously strengthening governance and civic participation mechanisms.

In practice, urban planners should:

- Prioritize ecological design that enhances not only physical green coverage but also citizen perception and usability.
- Promote shared space governance mechanisms that lower access barriers and enhance spatial equity.
- Facilitate spatial interaction through connected and multifunctional urban designs.
- Empower citizens through environmental education and participatory planning.

## Data Availability

The raw data supporting the conclusions of this article will be made available by the authors, without undue reservation.
