# Supplementary material for: How ecological, production, and living spaces jointly shape urban spatial integration through resource sharing and interaction
Source: Front Public Health. 2025 Sep 4;13:1651646. doi: 10.3389/fpubh.2025.1651646 (PMC12443693; doi:10.3389/fpubh.2025.1651646)
Supplement: Supplementary file 1 [file Table_1.docx]

Appendix Table 1 Cronbach’s Alpha Reliability Analysis

| **Construct** | **Item** | **Corrected Item-Total Correlation** | **Cronbach’s Alpha if Item Deleted** | **Cronbach’s Alpha** |
| --- | --- | --- | --- | --- |
| PSV | PSV1 | 0.768 | 0.885 | 0.907 |
|  | PSV2 | 0.754 | 0.888 |  |
|  | PSV3 | 0.769 | 0.885 |  |
|  | PSV4 | 0.777 | 0.884 |  |
|  | PSV5 | 0.757 | 0.888 |  |
| SLS | SLS1 | 0.721 | 0.876 | 0.895 |
|  | SLS2 | 0.689 | 0.881 |  |
|  | SLS3 | 0.73 | 0.875 |  |
|  | SLS4 | 0.728 | 0.875 |  |
|  | SLS5 | 0.694 | 0.88 |  |
|  | SLS6 | 0.747 | 0.872 |  |
| PQES | PQES1 | 0.732 | 0.845 | 0.878 |
|  | PQES2 | 0.748 | 0.839 |  |
|  | PQES3 | 0.734 | 0.845 |  |
|  | PQES4 | 0.733 | 0.845 |  |
| DSSR | DSSR1 | 0.724 | 0.831 | 0.869 |
|  | DSSR2 | 0.696 | 0.842 |  |
|  | DSSR3 | 0.744 | 0.823 |  |
|  | DSSR4 | 0.72 | 0.833 |  |
| FSI | FSI1 | 0.72 | 0.845 | 0.876 |
|  | FSI2 | 0.703 | 0.849 |  |
|  | FSI3 | 0.726 | 0.844 |  |
|  | FSI4 | 0.696 | 0.852 |  |
|  | FSI5 | 0.685 | 0.854 |  |
| EPPI | EPPI1 | 0.663 | 0.78 | 0.829 |
|  | EPPI2 | 0.677 | 0.774 |  |
|  | EPPI3 | 0.63 | 0.795 |  |
|  | EPPI4 | 0.652 | 0.785 |  |
| PEA | PEA1 | 0.699 | 0.808 | 0.851 |
|  | PEA2 | 0.663 | 0.822 |  |
|  | PEA3 | 0.698 | 0.807 |  |
|  | PEA4 | 0.706 | 0.804 |  |
| DUSI | DUSI1 | 0.728 | 0.862 | 0.886 |
|  | DUSI2 | 0.754 | 0.852 |  |
|  | DUSI3 | 0.748 | 0.854 |  |
|  | DUSI4 | 0.773 | 0.845 |  |
