# Supplementary material for: How ecological, production, and living spaces jointly shape urban spatial integration through resource sharing and interaction
Source: Front Public Health. 2025 Sep 4;13:1651646. doi: 10.3389/fpubh.2025.1651646 (PMC12443693; doi:10.3389/fpubh.2025.1651646)
Supplement: Supplementary file 2 [file Table_2.docx]

Appendix Table 2. Validity Test: KMO and Bartlett's Test of Sphericity

| **KMO Measure of Sampling Adequacy** | | **0.916** |
| --- | --- | --- |
| Bartlett's Test of Sphericity | Approximate Chi-Square | 14661.421 |
|  | Degrees of Freedom (df) | 630 |
|  | Significance Level (p-value) | 0.000 |
